# CRISPRminer is a knowledge base for exploring CRISPR-Cas systems in microbe and phage interactions

**DOI:** 10.1038/s42003-018-0184-6

**Published:** 2018-10-31

**Authors:** Fan Zhang, Shijia Zhao, Chunyan Ren, Yuwei Zhu, Haibin Zhou, Yongkui Lai, Fengxia Zhou, Yuqiang Jia, Kangjie Zheng, Zhiwei Huang

**Affiliations:** 1 HIT Center for Life Sciences, School of Life Science and Technology, Harbin Institute of Technology, 150080 Harbin, China; 2 School of Computer Science and Technology, Harbin Institute of Technology, 150080 Harbin, China; 3 Division of Hematology/Oncology, Boston Children’s Hospital, Harvard Medical School, Boston, MA 02115 USA

## Abstract

CRISPR-Cas systems not only play key roles in prokaryotic acquired immunity, but can also be adapted as powerful genome editing tools. Understanding the native role of CRISPR-Cas systems in providing adaptive immunity can lead to new CRISPR-based technologies. Here, we develop CRISPRminer, a knowledge base and web server to comprehensively collect and investigate the knowledge of CRISPR-Cas systems and generate instructive annotations, including CRISPR arrays and Cas protein annotation, CRISPR-Cas system classification, self-targeting events detection, microbe–phage interaction inference, and anti-CRISPR annotation. CRISPRminer is user-friendly and freely available at http://www.microbiome-bigdata.com/CRISPRminer.

## Abstract (web summary)

Fan Zhang et al. present CRISPRminer, a comprehensive database for exploring CRISPR-Cas systems in more than 3500 microbial species and a web-server for in-depth data mining. CRISPRminer allows researchers to predict CRISPR-Cas systems and investigate self-targeting CRISPRs, microbe-phage interaction networks, and anti-CRISPR features.

## Introduction

CRISPR (clustered regularly interspaced short palindromic repeats)-Cas (CRISPR-associated proteins) systems are prokaryotic adaptive immune systems that are known to protect bacteria and archaea from phage infection. Understanding the native role of CRISPR-Cas systems and their programmable nature is very inspiring for adaptive immune system studies and can lead to the development of new CRISPR-based technologies.

Bacteria and phages are engaged in an evolutionary arms race^1^. Bacteria utilize CRISPR-Cas adaptive immune systems for protection against phage infection^2^. In turn, some phages have been found capable to evade CRISPR-Cas immunity using anti-CRISPR proteins^3,4^. Several databases or web servers have been developed to gather information and provide useful applications to study CRISPR-Cas systems, such as CRISPRs web server (including CRISPRFinder^5^, CRISPRdb^6^, and CRISPRcompar^7^), CRISPRCasFinder^8^, CRISPRone^9^, CRISPI^10^, and anti-CRISPRdb database^11^. CRISPRFinder^5^ and its updated version CRISPRCasFinder^8^ are tools to detect CRISPR arrays and Cas gene clusters. CRISPRdb^6^ focus on displaying CRISPR arrays and generating dictionaries of spacers and repeats. CRISPRcompar^7^ is used to compare CRISPRs. In general, the CRISPRs web server, CRISPRCasFinder^8^, CRISPI^10^ and CRISPRone^9^, focuses on the annotation and characterization of CRISPR arrays of repeat-spacer units, and/or the Cas locus, while the anti-CRISPRdb^11^ is a database of manually collected anti-CRISPR proteins and their homologs from publications. However, each of these databases only focuses on a specific field of CRISPR biology and provides limited information concerning CRISPR-Cas systems or anti-CRISPRs, without building a connection between the microbe and phage based on CRISPR-Cas immunity. Currently, with the expansion of the diversity and classification of CRISPR-Cas systems, and novel findings on microbe–phage coevolution, the study of CRISPR-Cas systems requires all-inclusive data from multiple aspects/dimensions combined with thorough mining, yet such a comprehensive online resource or web server is still lacking.

Here, we develop the CRISPRminer database and web server to fill this gap by comprehensively collecting and mining the knowledge underlying these systems from published observations and generating instructive annotations, and developing pipelines for CRISPR-Cas system prediction, classification, and annotation based on python scripts and a series of public tools.

## Results

### Database content

**Fig. 1:**
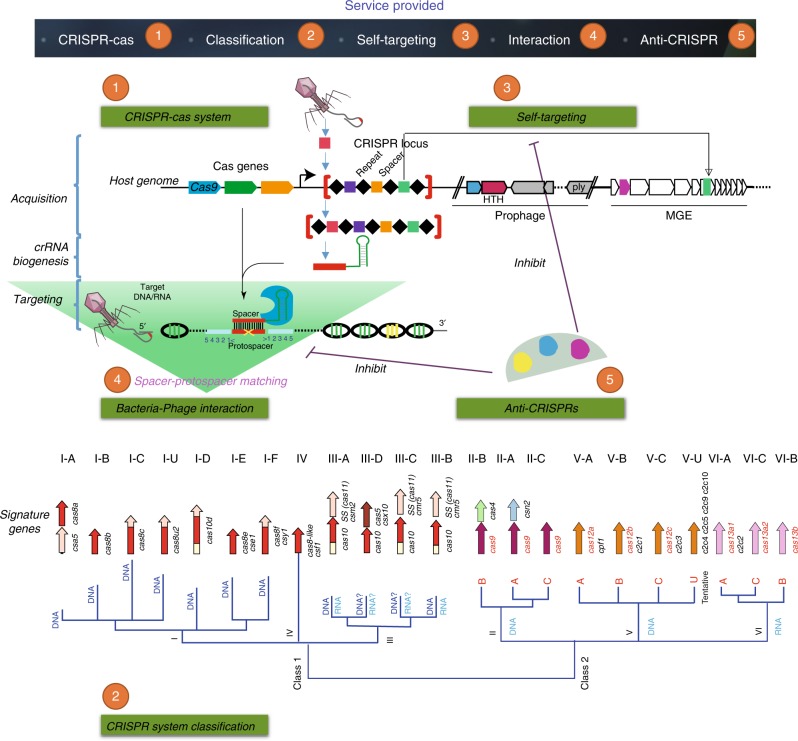
Five categories of information important for CRISPR studies are integrated in CRISPRminer database and the corresponding prediction services are also provided

The current CRISPRminer database has integrated five categories of information that are experimentally or computationally identified to be important for CRISPR studies (Fig. 1), including annotation and visualization of the CRISPR-Cas systems in 43,140 bacterial and 167 archaeal organisms from 3588 species (1279 genera), classification of the CRISPR-Cas systems into six types (21 subtypes) based on signature Cas genes and distinctive gene architectures, collection of 22,110 self-targeting events from publications and detection of 6260 putative self-targeting events in 4136 bacterial organisms, inference of putative 55,279 microbe−phage interactions based on CRISPR-spacer sequences and 1972 relations extracted from NCBI database, and annotation of the anti-CRISPR proteins experimentally identified in published papers.

### CRISPR array and Cas protein annotation module

**Fig. 2:**
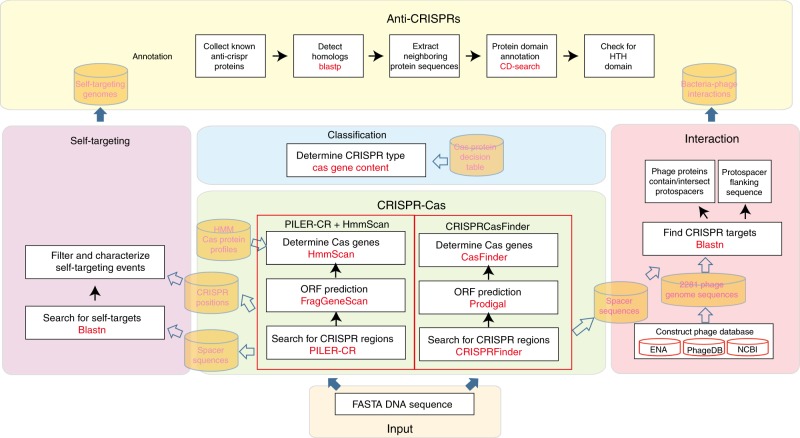
The flowchart of the prediction procedure and the overview of the prediction tools and the underlying data

By investigating completely or partially sequenced archaeal and bacterial genomes downloaded from the NCBI FTP site (ftp://ftp.ncbi.nlm.nih.gov/genomes/all/), we identified 91,685 CRISPR arrays in 62,176 bacterial and 167 archaeal organisms (distributed in 3588 species of 1279 genera) using PILER-CR^12^, among which 43,140 organisms (including 2467 complete genomes and 262 plasmids) are detected with both the CRISPR arrays and the corresponding Cas proteins. The process for CRISPR-Cas system detection is demonstrated in Fig. 2. Briefly, the PILER-CR^12^ program is used with default parameters to identify CRISPR arrays, and then sequences including 15 kbp upstream and downstream of the CRISPR arrays are extracted. Open Reading Frame (ORF) annotation is performed using FragGeneScan^13^. All ORFs are further annotated using RPS-BLAST against 395 profiles that represent 93 distinct Cas protein families. The CRISPR arrays lacking nearby Cas genes or Cas clusters lacking nearby CRISPRs are defined as isolated ones. The type of each CRISPR-Cas locus is determined according to the signature cas genes, such as major effector module genes including cas7, cas5, cas8, cas10, csf1, cas9, and cpf1^14^. At the genus level, *Mycobacterium*, *Escherichia*, *Clostridioides*, and *Salmonella* contribute to approximately 50% of the detected CRISPR systems. At the species level, *Mycobacterium tuberculosis*, *Escherichia coli*, *Clostridioides difficile*, and *Salmonella enterica* are the dominant species. However, the enriched genus and species may be due to the largest number of strains sequenced for health-related issues, but not the actual distribution of these CRISPR-Cas systems in the microbial world.

In the CRISPR-Cas module, users can browse the precalculated CRISPR-Cas systems through the taxonomy tree, or fuzzy search of the bacterial and archaeal description or using RefSeq accession numbers. Both global and local views are provided to visualize the detailed information. The global view provides the locations of the CRISPR regions on the genome coupled with the predicted genomic islands (GIs) using IslandViewer^15^ and predicted prophage regions using Prophinder^16^ and PHASTER^17^. The local view provides a visualization of the CRISPR arrays (shown as x10 for example, where 10 denotes the copy number of the repeats) and the Cas proteins (colored based on their functions such as acquisition, ancillary, CASCADE, crRNA maturation, and helper) with detailed information for the CRISPR array (location, repeats, and spacers) and Cas genes (locations, protein sequences, HMM profiles, and type/subtype). In addition, using the “compare” tool, users can compare the CRISPR systems among the organisms belonging to the same species or genus.

### CRISPR-Cas system classification module

The Classification module provides (sub) types annotation of the CRISPR-Cas systems based on the signature genes and distinctive gene architectures according to the most recent classification studies^18,19^ (Fig. 2). The classification scheme encompasses two classes and six types. Class 1 CRISPR-Cas systems are defined by the presence of a multi-subunit crRNA–effector complex, including type I (I-A, I-B, I-C, I-D, I-E, I-F, and I-U subtypes) and type III (III-A, III-B, III-C, and III-U subtypes) CRISPR-Cas systems, as well as the putative new type IV^20^. Class 2 CRISPR-Cas systems are defined by the presence of a single subunit crRNA–effector module, including type II (II-A, II-B, and II-C subtypes), as well as two putative new types V (V-A, V-B, V-C, and V-U subtypes) and VI (VI-A, VI-B, and VI-U subtypes)^21^. Users can conveniently browse the bacterial organisms belonging to each (sub) type with detailed information, such as bacterial description, RefSeq ID, Genbank ID, and the Cas operon composition.

### Self-targeting module

Self-targeting is suggested as a form of autoimmunity^22^, and has been used as a genomic marker to screen CRISPR-Cas inhibitor genes in Listeria monocytogenes^23^. The Self-Targeting module includes 22,110 self-targeting events collected from publications^22–24^ and 6260 putative self-targeting events detected from 4136 CRISPR containing organisms using a similar methodology to previous reports^13^ (Fig. 2). Briefly, self-targeting spacers are detected using blastn (with default settings and an e-value limit of 10e−2) against all the contigs within the same assembly except for the DNA bases within the predicted CRISPR arrays. These published and putative self-targeting events will enable the further exploration of their biological significance in CRISPR-based autoimmunity.

### Microbe−phage interaction module

Recent work suggests that phage−host interactions can be inferred by analyzing CRISPR spacer sequences based on the hypothesis that hosts only incorporate spacer sequences from the phages that infect them^25^. In the “Interaction” module, to infer a large-scale microbe−phage infection network, the spacers from each microbe organism is collected and used to BLAST (blastn with parameters settings: word_size = 8, e-value < 0.01, and up to three mismatches allowed) against an integrated database of 2281 phage and archaeal virus genomes from the NCBI viral Genome Resource, the EMBL EBI phage genomes and the phagedb databases (Fig. 2). The interactions are grouped by their host and could be easily browsed or indexed by their host names. Additionally, information for interacting spacers and their targeting proto-spacers, as well as the phage genome content near proto-spacers, is provided. If the proto-spacer is located in a protein region, the protein annotation is also provided. Finally, each phage genome is also searched for its bacterial or archaeal host, which is annotated under the fields “isolate host=” or “host=” in its Genbank file. This analysis could help establish the link between phages and microbe, which might be an important supplement to the current understanding of natural microbial communities.

### Anti-CRISPRs annotation module

This module contains all 23 anti-CRISPR protein families detected and validated in the CRISPR-bearing bacterial or archaeal organisms, including four from type I-E, ten from type I-F, five from type II-A, three from type II-C, and one from type I-D. Since more anti-CRISPRs are estimated to be uncovered, we searched for homologs using blastp (with e-value < 0.01, identity > 40%) of all validated anti-CRISPR proteins, extracted the surrounding genomic content (five proteins upstream and downstream, respectively), and determined their functions using rpsblast with an e-value cutoff of 10e−2 against the Conserved Domain Database (CDD)^26^. The operons encoding anti-CRISPR proteins could be studied for further identification of conserved characteristics that are useful for anti-CRISPR prediction, such as Aca (anti-CRISPR-associated) genes with the HTH (helix-turn-helix) domain usually located downstream of the anti-CRISPRs (Fig. 2).

### Prediction module

The Prediction module is used to annotate CRISPR-related knowledge (including CRISPR arrays and Cas clusters annotation, CRISPR-Cas systems classification, interacting phages inference, self-targeting events detection, and anti-CRISPR homolog searching) for an existing or a newly sequenced genome by directly uploading or pasting the nucleotide sequence in FASTA format. The prediction procedure underlying CRISPRminer is implemented using python scripts and a series of public tools including blastn/blastp, rpsblast, HmmScan, FragGeneScan, PILER-CR, and CRISPRCasFinder (Fig. 2). At the CRISPR-Cas system annotation step, both the PILER-CR+HmmScan and CRISPRCasFinder strategies are supported. Briefly, in CRISPRCasFinder procedure, the CRISPR arrays are identified using CRISPRFinder, putative ORFs are identified using Prodigal v2.6.3, cas gene clusters and their (sub)type are determined using MacSyFinder. The detected CRISPR arrays coupled with nearby (within 10kbp) Cas clusters are identified as CRISPR-Cas loci according to their distances using the dynamic programming algorithm. The PILER-CR+HmmScan procedure is the same as previously described in CRISPR-Cas system annotation module.

### Web server

CRISPRminer is implemented using the ThinkPHP framework and the front-end design is created using the Bootstrap framework, which is a well-established tool for intuitive design. Besides, the front-end design is also created using the cross-platform JavaScript library jQuery, which is designed to simplify the client-side scripting of HTML. In addition, CRISPRminer also uses DataTable, the plugin for jQuery, to conveniently display data. CRISPRminer back-end runs on top of an Apache server, and the communication with the server is made through a Common Gateway Interface. The server works by redirecting or sending the user input to the python script for handling and displaying the results in numerical, textual, and visual output.

## Discussion

In summary, we comprehensively collected information about CRISPR-Cas systems and anti-CRISPRs to construct the CRISPRminer web server, seeking to accelerate our understanding of the co-evolutionary relationship between microbe and phages. As a specifically designed CRISPR knowledge database, CRISPRminer not only integrates the dispersed data but also provides a convenient way for in-depth data mining. First, we provide a Prediction module to investigate all five categories of CRISPR-related information for an existing or a newly sequenced genome by uploading or pasting the sequence. Second, we provide several techniques for browsing and comparing the detected CRISPR-Cas systems in the CRISPR-Cas module. Users can look up desired bacterial and archaeal genomes through the taxonomy tree, or by a fuzzy search of the microbial description or use of the RefSeq accession number. The CRISPR systems could also be compared at different taxonomy levels (species or genus) and coupled to the annotation of genomic islands (GIs, regions of probable horizontal origin) and prophage regions. Third, by studying self-targeting spacers from all detected CRISPR arrays in currently sequenced bacterial genomes, users could further explore their biological meanings in CRISPR-based autoimmunity. Fourth, by blasting 918,168 spacers against an integrated database of 2281 phage genomes, a large-scale microbe–phage infection network is inferred and could support further characterization of the interaction patterns and underlying processes. Finally, we incorporated experimentally validated anti-CRISPR proteins and their homologs, and annotated their surrounding gene content to facilitate anti-CRISPR discovery. This comprehensive and user-friendly CRISPRminer offers a large collection of information that could be useful to the CRISPR or microbiology community and is a much-needed tool that will facilitate research on CRISPR biology and complement other available tools for CRISPR-based applications such as genome editing. Our database will be updated regularly with newly released bacterial and archaeal genomes and provide all five categories of CRISPR-related information for these genomes automatically by utilizing the underlying prediction procedure.

### Code availability

The public tools used in this study can be downloaded from their official website, such as blastn/blastp and rpsblastp (https://blast.ncbi.nlm.nih.gov/Blast.cgi), HmmScan (https://www.ebi.ac.uk/Tools/hmmer/search/hmmscan), PILER-CR (https://www.drive5.com/pilercr/), FragGeneScan (http://omics.informatics.indiana.edu/FragGeneScan/), and CRISPRCasFinder (https://crisprcas.i2bc.paris-saclay.fr/Home/Download). CRISPRminer user manual is available at http://www.microbiome-bigdata.com/CRISPRminer/index.php/Home/Index/help. The source code for CRISPRminer is available in the GitHub repository (https://github.com/hitzhangfan/microbiome-bigdata-Analyzer/tree/master/CRISPRminer)

## Data Availability

Bacterial and archaeal genome sequences were downloaded from NCBI FTP site (ftp://ftp.ncbi.nlm.nih.gov/genomes/all/) in May 2017. 395 Cas profiles were collected from a recent study (Makarova et al., Nat. Rev. Microbiol. (2015)). Prophage regions were extracted from PHASTER (http://phaster.ca/) and ACLAME database (http://aclame.ulb.ac.be/Tools/Prophinder/). Genome islands were downloaded from IslandViewer 4 web server (http://www.pathogenomics.sfu.ca/islandviewer/). Phage genomes were downloaded from NCBI viral Genome Resource (https://www.ncbi.nlm.nih.gov/genome/viruses/), EMBL EBI phage genomes (https://www.ebi.ac.uk/genomes/phage.html), and phagedb (http://phagesdb.org/). Besides, the datasets generated and/or analyzed during the current study could be downloaded online from the CRISPRminer website (http://www.microbiome-bigdata.com/CRISPRminer/). We confirm that all relevant data are available from the authors.
